# HIV, syphilis and hepatitis B coinfections in Mkushi, Zambia: a cross-sectional study

**DOI:** 10.12688/f1000research.17983.2

**Published:** 2020-07-27

**Authors:** Cibangu Katamba, Theresa Chungu, Chisali Lusale

**Affiliations:** 1 ART Department, Mkushi District Hospital, Mkushi, Zambia; 2 Administration Department, Mkushi District Hospital, Mkushi, Zambia

**Keywords:** HIV, Hepatitis B, Syphilis, Coinfections

## Abstract

**ABSTRACT**

**Background:** Human Immunodeficiency Virus, syphilis and Hepatitis B Virus are major global public health problems, they are sexually transmitted infections with overlapping modes of transmission and affected populations.

**Objective:** The aim of this study is to assess the seroprevalence of HIV 1, hepatitis B virus and syphilis coinfections among newly diagnosed HIV individuals aged 16 to 65 years, initiating on antiretroviral therapy, in Mkushi, Zambia.

**Methods:** A total number of 126 sera were collected from HIV 1 infected patients attending Mkushi district hospital/ART clinic for antiretroviral therapy initiation. Hepatitis B surface antigen test and serologic test for syphilis were conducted between March and May 2018.

**Results:** Of the 126 participants (out of 131 enrollments), Hepatitis B surface antigen (HBsAg) was detected with a prevalence of 9.5% among newly diagnosed HIV infected patients, while that of syphilis was as high as 40.5% in this same population group. Three patients recorded HIV coinfections with both syphilis and hepatitis B virus (2.4%) at the same time. After analysis, the results indicate that there was no significant association between gender for both dependent variables: HIV/syphilis or HIV/hepatitis B virus coinfections (alpha significance level > 0.05). Those who had a history of syphilis infection in the past were more likely than those who had none to be HIV-syphilis coinfected (53.6% vs 34%, respectively; odd ratio [OR] 2.236; 95% confidence interval [CI] 1.045 – 4.782).

**Conclusion:** The high prevalence rates for HIV, HBV, and syphilis coinfections strongly indicate the need for HBV and syphilis screening for HIV infected individuals. Furthermore, the high number of patients previously treated for syphilis who retest positive for syphilis in this study calls for use of the Venereal Disease Research Laboratory test to identify true syphilis infection (titers ≥ 1:8 dilutions, strongly suggestive).

## Introduction

Hepatitis B virus, together with syphilis and human immunodeficiency virus (HIV), are important public health challenges worldwide ^1^, they are transmitted through sex (Sexual transmitted infections, STIs) ^2– 5^ and cross over affected populations ^1^. The World Health Organization (WHO) reported an estimated 36.9 million people were living with HIV in 2016 ^6, 7^. It was also reported that 248 million had chronic Hepatitis B virus (HBV) infection (persistent HBs Ag ≥ six months) ^8^.

It is estimated that about a million sexually transmitted infections are acquired daily throughout the world ^5^. Moreover, STDs have impact on neonatal, sexual and reproductive health. They can also cause severe complications if not treated. Additionally, STIs pose major socioeconomic challenge on treatment costs ^5, 9, 10^. Many studies have demonstrated a two-sided relationship between HIV and a number of STIs, including syphilis and HBV ^4^. Syphilis and HBV has the potential to increase genital and plasma HIV 1 RNA levels. This increase can raise the likelihood of transmission of HIV 1 to others. In the same way, HIV 1 infection has the potential to impact the clinical manifestation, the therapeutic outcome and the HBV/syphilis disease progression ^4^.

HIV is associated with a higher prevalence of both HBV and Hepatitis C Virus (HCV) in Sub-Saharan Africa ^11– 13^ in this region, many people living with HIV are HBV or HCV coinfected. About 8% of individuals infected with HIV were HBV coinfected in another study conducted in this location ^14^. In a trial conducted in Sub-Saharan Africa ^15^, a number of factors were associated with high rates of HIV and syphilis coinfection, including: young age, divorce, widowhood, or separation. Although it is helpful to recognize syphilis risk factors, many women without these characteristics were syphilis-seroreactive ^15^. Also, a high proportion of prevalent rapid plasminogen reagin (RPR)-positive infections remain serofast despite treatment ^16^.

In a number of studies conducted in Zambia and the region ^17– 29^, it has been observed that HIV and chronic hepatitis B (CHB) coinfection was common in both children and adults. It was also observed in these patients that many risk factors such as liver complications and impaired immunologic recovery increased morbidity and mortality. In these settings, syphilis infection remains prevalent in HIV infected adults ^30^. As HIV, syphilis and HBV coinfections are clinically consequential, there is great need to screen for syphilis and HBV in people living with HIV/AIDS. The aim of this study was to determine the prevalence of HIV1, HBV and Treponema pallidum serological markers, and therefore their coinfection prevalence among newly diagnosed HIV individuals attending ART clinic in Mkushi rural district of Zambia. The lack of current prevalence estimates of HIV/HBV and/or syphilis co-infections in Mkushi provides the rationale for this project. Also, laboratory based diagnostic tests for the three infections are not routinely conducted together in much of Zambia.

## Methods

### Ethical considerations

This study was conducted after obtaining permission from the Mkushi District Health Office. The waiver for ethical clearance was sought and obtained from the ERES Converge Zambian Institutional Review Board (IRB); the authority to publish was sought and obtained from the Zambian National Health Research Authority (NHRA). Participants were informed and provided verbal consent prior to enrollment in the study.

### Study design

We performed a cross-sectional study to assess the seroprevalence of HIV 1, HBV and syphilis coinfections among newly diagnosed HIV individuals aged 16 to 65 years, initiating on cART, in Mkushi, Zambia. Sociodemographic and clinical data were collected from study subjects including age, sex, number of years of education, marital status, residence, occupation, household income, HIV transmission route, history of tuberculosis, past syphilis infection, and WHO clinical staging. A data collection sheet (Extended data ^31^) was used to collect information in addition to client’ files. The study was conducted by a team of trained medical officers, nurses and laboratory technicians.

Inclusion and exclusion criteria: this study included a patient population starting ART between March and May 2018, aged 16 to 65 years old, confirmed HIV 1 infection, being ART naïve (never taken ART for their HIV infection), regardless of WHO clinical staging. Subjects previously exposed to ART, children below the age of 16, and clients with HIV 2 or HIV 1 & 2 coinfections were excluded. A total number of 126 subjects were included.

Diagnosis: All tests were performed at Mkushi hospital laboratory. Four milliliters of venous blood were collected from each client using aseptic technic. Following centrifugation, sera were separated and tested immediately for HIV antibodies. HIV serologic test was conducted using Determine screening test (ALERE) (CHASE BUFFER REF 7D2243, ALERE Medical Co. Ltd. Japan); followed by SD Bioline confirmatory test, discriminating between HIV 1 & HIV 2. (SD HIV1/2 3.0) (Assay diluent Lot 03ADDC013, SD Standard diagnostics, Inc. Hepatitis B virus infection was assessed by an immunochromatographic test (one step rapid test, Lot No. HBPDR00117, NeckLife, Punjab), following the manufacturer’s instructions. Syphilis serology was examined using rapid plasma reagin (RPR) (one Step Rapid Test, Lot No. SYS-02-18Q, Ensure Biotech, Hyderabad-500 076), following the manufacturer’s instructions.

Statistical analysis: The collected data were analyzed using the IBM SPSS statistics software (trial version 20). The Chi-Square test was used for analysis of the differences between proportions and p < 0.05 was considered as significant. The statistical analysis of frequencies was also conducted and the confidence interval was set at 95%.

## Results

Of the 126 participants (out of 131 enrollments in the same category), 46 were male (36.5%) and 80 were female (63.5%), 102 were between the age of 19 and 40 years excluded (81%), 100 had primary education (79.4%). While 66 patients were married (52.4%), the rest were either divorced (30/126), never married (22/126) or widowed (8/126).

A total number of 126 blood samples were collected from HIV 1 infected patients attending Mkushi district hospital/ART clinic for antiretroviral therapy initiation (see underlying data ^24^). HIV 1, HBV, and Syphilis coinfections were recorded together in 2.4% of cases (3/126).

Table 1 shows the prevalence of syphilis coinfection with HIV by age group.

**Table 1.** Positive syphilis serostatus by age group.

Current syphilis by age group cross tabulation						
Count						
		Age group				Total
		< 20	20 – 29 years	30 – 39 years	40 and above	
Current Syphilis	Positive	1	24	15	11	51
	Negative	3	36	27	9	75
Total		4	60	42	20	126

The overall syphilis seroprevalence was 40.5% (51/126) in the studied population. HIV infected patients in the age group 20 – 29 years recorded the highest syphilis prevalence 47% (24/51), followed by the age group 30 – 39 years at 29% (15/51), while those in the age group below 20 years recorded the lowest prevalence of 2% (1/51).

The below table shows the hepatitis B virus and syphilis coinfections results by gender among HIV infected subjects ( Table 2).

**Table 2.** HIV, HBV and syphilis coinfections.

Hepatitis B Virus			
	Male	Female	Total
**Reactive**	4	8	12
**Non-reactive**	42	72	114
**Current Syphilis**			
**Reactive**	17	34	51
**Non-reactive**	29	46	75

Hepatitis B surface antigen (HBsAg) was detected with a prevalence of 9.5% (12/126) among newly diagnosed HIV infected patients, while that of syphilis was as high as 40.5% (51/126) in this same population group. The above data was analyzed and the results indicate that there was no significant association between gender and syphilis (Chi square value = 0.373, p = 0.542). This study also shows that HBV presence was independent of gender (alpha significance level > 0.05).

A past medical history was taken from each individual to obtain information on past syphilis infection. The results of past and current syphilis are shown in the table below ( Table 3).

**Table 3.** Past vs current syphilis risk estimate.

Past syphilis by current syphilis crosstabulation				
Count				
		Current Syphilis		Total
		Positive	Negative	
Past Syphilis	Positive	22	19	41
	Negative	29	56	85
Total		51	75	126

Finally, those HIV clients who had a history of syphilis infection in the past were more likely than those who had none to be HIV-syphilis coinfected (53.6% vs 34%, respectively; OR 2.236; 95% CI 1.045 – 4.782). The relative Odds of HIV-Syphilis coinfection is calculated at 1.573 (95% CI: 1.044 – 2.370).

## Discussion and conclusion

The correlates of HIV, syphilis and hepatitis B coinfections were age (between 20 and 39 years), primary education, and of course sexual activity. This may be influenced by the rural settings where our study was conducted (especially low level of education). The HBV/HIV coinfections prevalence of 9.5% in this study is in line with the prevalence of 9.9% documented in Zambia by Kapembwa and colleagues ^27^, and also the 12.2 reported by Vinikoor *et al*. ^19^. The later also demonstrated that HIV coinfected adult males were more likely to be coinfected with HBV than their female counterparts. This contrast to our findings may be due to our small sample size. In our study, Pearson Chi-square analysis showed no statistically significant difference between gender for both HIV/HBV and HIV/Syphilis coinfections. Our findings demonstrate that HIV infected clients are more likely to be infected with other STIs, especially syphilis. The coinfection prevalence of 40.5% for HIV and syphilis agrees with previous study conducted in Zambia (43%) by Odom and colleagues ^16^. We found past syphilis infection to have positive association with two-fold increase risk of HIV/syphilis coinfections.

Limitations: The difference in performance observed with various hepatitis, syphilis, and HIV diagnostic test kits available in the market might influence the comparison of results with other studies. Moreover, this facility based survey may limit the generalizability of findings to all people living with HIV due to possible selection and information bias.

However, as shown earlier, the occurrence of these coinfections is clinically consequential. The information provided in this study will therefore assist policy makers re-direct resources for concomitant screenings/diagnosis of HIV, HBV, and syphilis.

CONCLUSION: The high prevalence rates for HIV, HBV, and syphilis coinfections strongly indicate the need for HBV and syphilis screening for HIV infected individuals. The 2018 Zambian consolidated guidelines for prevention and treatment of HIV infection address the management of these coinfections ^32^. However, there is urgent need to monitor the implementation of HBV and syphilis testing among HIV infected subject to close the gap between policy and practice. Furthermore, in previous study conducted in Zambia, Odom and colleagues ^16^ demonstrated that a high proportion of RPR positive infections remain serofast despite treatment. The high number of patients previously treated for syphilis who retest positive for syphilis in this study calls for use of the Venereal Disease Research Laboratory test to identify true syphilis infection (titers ≥ 1:8 dilutions, strongly suggestive) ^33^. Both government and Non-governmental organizations need to up their efforts to support these health care service needs appropriately.

## Data availability

### Underlying data

Harvard Dataverse: Replication Data for: HIV, SYPHILIS AND HEPATITIS B COINFECTIONS IN MKUSHI RURAL, ZAMBIA: a cross-sectional study. https://doi.org/10.7910/DVN/AK3JZR ^34^

This project contains the following underlying data:

- HIV_HBV_SYPHILIS.tab (Seroprevalence data)

### Extended data

Harvard Dataverse: Cibangu, Katamba, 2019, "Replication Data for: HIV, SYPHILIS AND HEPATITIS B COINFECTIONS IN MKUSHI RURAL, ZAMBIA: a cross-sectional study". https://doi.org/10.7910/DVN/6SNMQS ^31^

This project contains the following extended data:

- Data collection sheet.docx (data collection sheet)
