# Supplementary material for: HIV, syphilis and hepatitis B coinfections in Mkushi, Zambia: a cross-sectional study
Source: F1000Res. 2020 Jul 27;8:562. Originally published 2019 Apr 26. [Version 2] doi: 10.12688/f1000research.17983.2 (PMC7417957; doi:10.12688/f1000research.17983.2)
Supplement: Peer review history

# Peer review: Reviewer response for version 2

**DOI:** 10.5256/f1000research.28200.r68041

**Published:** 2020-08-10

**Authors:** Carolyn Bolton Moore

**Affiliations:** 1 Centre for Infectious Diseases Research in Zambia, Lusaka, Zambia; 2 University of Alabama at Birmingham, Birmingham, AL, USA

I am happy with the revised manuscript and have no additional comments or changes.

Is the work clearly and accurately presented and does it cite the current literature?

Partly

If applicable, is the statistical analysis and its interpretation appropriate?

Yes

Are all the source data underlying the results available to ensure full reproducibility?

Partly

Is the study design appropriate and is the work technically sound?

Yes

Are the conclusions drawn adequately supported by the results?

Yes

Are sufficient details of methods and analysis provided to allow replication by others?

Yes

Reviewer Expertise:

NA

I confirm that I have read this submission and believe that I have an appropriate level of expertise to confirm that it is of an acceptable scientific standard.

# Peer review: Reviewer response for version 2

**DOI:** 10.5256/f1000research.28200.r68042

**Published:** 2020-07-28

**Authors:** Sanika Chirwa

**Affiliations:** 1 Department of Neuroscience & Pharmacology, Meharry Medical College, Nashville, TN, USA; 2 Adjunct, Department of Pharmacology, Vanderbilt University, Nashville, TN, USA

The authors have adequately addressed all the concerns that were raised in the initial review.

Is the work clearly and accurately presented and does it cite the current literature?

Partly

If applicable, is the statistical analysis and its interpretation appropriate?

Partly

Are all the source data underlying the results available to ensure full reproducibility?

Partly

Is the study design appropriate and is the work technically sound?

Partly

Are the conclusions drawn adequately supported by the results?

Partly

Are sufficient details of methods and analysis provided to allow replication by others?

No

Reviewer Expertise:

Neuroscience & Clinical Pharmacologist - actively engaged in both basic and clinical research. Later includes focus on patient-centered care of HIV continuum of care with a focus on pediatric HIV.

I confirm that I have read this submission and believe that I have an appropriate level of expertise to confirm that it is of an acceptable scientific standard.

# Peer review: Reviewer response for version 1

**DOI:** 10.5256/f1000research.19668.r64070

**Published:** 2020-06-23

**Authors:** Carolyn Bolton Moore

**Affiliations:** 1 Centre for Infectious Diseases Research in Zambia, Lusaka, Zambia; 2 University of Alabama at Birmingham, Birmingham, AL, USA

Thank you for the opportunity to review this manuscript. Overall the manuscript is sound but there are several grammatical errors and some sentences that do not make sense eg the reference to the National Guidelines. Additionally, there does not seem to be ethical approval. My understanding is that Mkushi District Health Office is the administrative body that falls under the Ministry of Health and manages the public health services in that area. Unless they have a special mandate, of which I am not aware, they are not authorized to provide Ethical Clearance as they are not considered a regulatory body. They are, however, required to give permission for research to occur in their facilities. Given that this study involves human subjects, I recommend getting retrospective ethics approval if not yet obtained or, if it has been obtained, including this in the manuscript from a recognised regulatory authority.  Also, please include the total number of patients who were seen during the same period as the enrolment (ie the denominator), it is not clear what proportion of patients who were seen, enrolled in the study and had a specimen drawn. Please also check citations- the paragraph that  starts with ... in Zambia (17-29) includes references that are regional but not necessarily in Zambia. Lastly, I suggest using STI instead of STD as it appears many of these infections were subclinical.

Is the work clearly and accurately presented and does it cite the current literature?

Partly

If applicable, is the statistical analysis and its interpretation appropriate?

Yes

Are all the source data underlying the results available to ensure full reproducibility?

Partly

Is the study design appropriate and is the work technically sound?

Yes

Are the conclusions drawn adequately supported by the results?

Yes

Are sufficient details of methods and analysis provided to allow replication by others?

Yes

Reviewer Expertise:

Infectious Disease in Zambia. Paediatric HIV. Epidemiology.

I confirm that I have read this submission and believe that I have an appropriate level of expertise to confirm that it is of an acceptable scientific standard, however I have significant reservations, as outlined above.

# Response

**Published:** 2020-07-06

**Authors:** Cibangu Katamba

**Affiliations:** Zambian Ministry of Health, Zambia

Thank you very much for reviewing this manuscript.

 The grammatical errors have been revised and the new version will be availed soon.

 Waiver for ethical clearance was sought and obtained from the ERES Converge Zambian Institutional Review Board (IRB), the authority to publish was sought and obtained from the Zambian National Health Research Authority (NHRA). This will also appear in the new version.

 The total number of patients who were seen during this period has also been included as the enrollment. Citations have been checked and corrected accordingly. The term STI has been used instead of STD.

# Peer review: Reviewer response for version 1

**DOI:** 10.5256/f1000research.19668.r58393

**Published:** 2020-02-03

**Authors:** Sanika Chirwa

**Affiliations:** 1 Department of Neuroscience & Pharmacology, Meharry Medical College, Nashville, TN, USA; 2 Adjunct, Department of Pharmacology, Vanderbilt University, Nashville, TN, USA

There is great potential in the presented study in terms of highlighting HIV + HBV + syphilis comorbid profile within rural settings in Zambia. This information could guide the distribution of pertinent resources needed to curtail the infections while identifying gaps in the drive to end the HIV epidemic. However, the manuscript is not written to best advantage and will require significant revisions to be impactful. Within this context, the authors may wish to consider the following concerns.

 INTRODUCTION

- This has accurate information but fails to specifically indicate relevance and need for study. What are the current prevalence estimates for HIV/HBV and/or syphilis co-infections in Mkushi? in Zambia? Is this information lacking and, therefore, partly formed the basis for the research study?
- Are laboratory-based screening and/or diagnostic tests for the three infections routinely conducted together in much of Zambia, or this is lacking and provided another impetus for the study?

 METHODS

- Clarify if the “Mkushi District Health Office” has formal authority to independently review and give ethical approval to conduct clinical research studies consistent with the norm.
- Indicate how study participants were informed of study, and how (and who) conducted the enrollment into the study.
- State what percentage of new HIV cases initiated on cART did the reported 126 patients (i.e., enrolled in study between March and May 2018) reflect.
- It is unclear how many 4-mL venous blood draws were carried out on each patient. Were these blood draws separate from routine blood draws for patient care at the hospital?
- Provide more information on how the screening/diagnostics tests performed actually work. Indicate their limitations and/or likelihood of false-positives. For example, Rapid Plasma Reagin for syphilis detects nonspecific antibodies produced by the body while fighting other infections like Lyme disease. How was this checked in study?

 RESULTS

- Presented data are scanty and lack details. The authors are encouraged to expand this section and give additional information as well as correct errors in counts.

- Consider reporting socioeconomic and demographic characteristics of participants in Table format. Give values as mean ± STD here and elsewhere, accordingly.
- Stated syphilis seroprevalence is 51 of 126 cases. This is then subdivided into age groups, i.e., 1 for below 20 years; 24 for 20–29 years; and 15 for 30 – 39 years. But this yields a total of 40 and not 51 cases. What happened to the missing 11 patients?
- Like with syphilis, should also report “seroprevalence” data for HIV and HBV both by age and gender. Authors need to be consistent accordingly.
- Need clarification on the reliability of the collection of “past syphilis infection” history. How exactly was this done?
- More rigorous statistical analyses needed to properly interpret data and show significance as applicable. The significance of findings are unconvincing (and lost) as reported.

 DISCUSSION

- Interpretation and significance of findings are unconvincing. It is difficult to make an independent assessment since presented data lack much detail.
- Need to explain what gaps in knowledge have been filled by the study. For example, how will this information assist with patient care and/or re-direction of resources for concomitant screenings/diagnosis of HIV, HBV, and syphilis?
- Are the above services readily available in all health centers in Zambia, or there is a disparity between urban and rural health centers? Explain.
- Need to discuss the limitations of the research study.

Is the work clearly and accurately presented and does it cite the current literature?

Partly

If applicable, is the statistical analysis and its interpretation appropriate?

Partly

Are all the source data underlying the results available to ensure full reproducibility?

Partly

Is the study design appropriate and is the work technically sound?

Partly

Are the conclusions drawn adequately supported by the results?

Partly

Are sufficient details of methods and analysis provided to allow replication by others?

No

Reviewer Expertise:

Neuroscience & Clinical Pharmacologist - actively engaged in both basic and clinical research. Later includes focus on patient-centered care of HIV continuum of care with a focus on pediatric HIV.

I confirm that I have read this submission and believe that I have an appropriate level of expertise to confirm that it is of an acceptable scientific standard, however I have significant reservations, as outlined above.

# Response

**Published:** 2020-07-06

**Authors:** Cibangu Katamba

**Affiliations:** Zambian Ministry of Health, Zambia

Thank you very much for reviewing this manuscript.

 The relevance and need for the study have been included. The number of participants informed of the study and how/ who conducted the study have been described. The 4 Ml venous blood draws were the routine blood draws for ART clients base line tests as recommended by the national guidelines. Limitation of screening diagnostic tests have been described. Data errors have been corrected. The total number of patients who were seen during this period has also been included as the enrollment. Citations have been checked and corrected accordingly.

 The waiver for ethical clearance was sought and obtained from the ERES Converge Zambian Institutional Review Board (IRB), the authority to publish was sought and obtained from the Zambian National Health Research Authority (NHRA). This will also appear in the new version. The limitations of the research have also been discussed.
